# A Systematic Review on Socioeconomic Differences in the Association between the Food Environment and Dietary Behaviors

**DOI:** 10.3390/nu11092215

**Published:** 2019-09-13

**Authors:** Joreintje D. Mackenbach, Kyra G. M. Nelissen, S. Coosje Dijkstra, Maartje P. Poelman, Joost G. Daams, Julianna B. Leijssen, Mary Nicolaou

**Affiliations:** 1 Department of Epidemiology and Biostatistics, Amsterdam Public Health Research Institute, Amsterdam UMC, Vrije Universiteit Amsterdam, 1081HV Amsterdam, The Netherlands; 2 Department of Public Health, Amsterdam Public Health Research Institute, Amsterdam UMC, University of Amsterdam, 1105AZ Amsterdam, The Netherlands; 3 Department of Health Sciences, Vrije Universiteit Amsterdam, Amsterdam Public Health Research Institute, 1081HV Amsterdam, The Netherlands; 4 Department of Human Geography and Spatial Planning, Faculty of Geosciences, Utrecht University, 3584CB Utrecht, The Netherlands; 5 Medical Library, Amsterdam UMC, University of Amsterdam, 1105AZ Amsterdam, The Netherlands

**Keywords:** dietary intake, effect modification, food prices, food retailers, interaction, SES, socio-economic position

## Abstract

Little is known about socioeconomic differences in the association between the food environment and dietary behavior. We systematically reviewed four databases for original studies conducted in adolescents and adults. Food environments were defined as all objective and perceived aspects of the physical and economic food environment outside the home. The 43 included studies were diverse in the measures used to define the food environment, socioeconomic position (SEP) and dietary behavior, as well as in their results. Based on studies investigating the economic (n = 6) and school food environment (n = 4), somewhat consistent evidence suggests that low SEP individuals are more responsive to changes in food prices and benefit more from healthy options in the school food environment. Evidence for different effects of availability of foods and objectively measured access, proximity and quality of food stores on dietary behavior across SEP groups was inconsistent. In conclusion, there was no clear evidence for socioeconomic differences in the association between food environments and dietary behavior, although a limited number of studies focusing on economic and school food environments generally observed stronger associations in low SEP populations. (Prospero registration: CRD42017073587)

## 1. Introduction

Socioeconomic inequalities in dietary behavior are persistent and widespread [1] and are contributing to inequalities in diet-related chronic diseases [2]. Several explanatory mechanisms for these inequalities have been proposed. Individuals with lower socioeconomic position (SEP) according to educational attainment, income levels or occupation status may lack the material and psychosocial resources that generally accompany a higher SEP. Indeed, material resources such as higher food budgets and access to health-promoting goods and services [3,4] and psychosocial resources such as nutrition knowledge, cooking skills and positive attitudes towards healthy eating [5,6,7,8] are known to contribute to healthier dietary behavior. 

Having fewer material and psychosocial resources may limit individuals’ capacity to resist unhealthy temptations in the food environment [9] or to take advantage of healthy options in the food environment. For example, higher educated individuals may be better able to deal with an unhealthy food environment because of their individual-level resources such as higher food budgets, better planning skills or more nutritional knowledge compared to those with lower education levels. If food environments are characterized by the availability and promotion of high-energy and ultra-processed foods—as is common in most Western countries [10]—food choices of those having fewer material and psychosocial resources are more likely to be unhealthy. 

While there is some evidence that food environments are unhealthier in more deprived areas [11]—also referred to as the ‘deprivation amplification’ or the double burden of deprivation [12,13]—little is known about the differential effects of the food environment on dietary behavior in higher and lower SEP groups. Such socioeconomic inequalities in the effects of the food environment on dietary behavior could in fact provide an explanation for the weak or inconsistent associations described in the numerous systematic literature reviews summarizing the influence of the food environment on dietary behavior so far [14,15,16,17,18,19,20,21,22,23,24]. If individuals with a high or low SEP respond differently to their food environments, this may confound the association between aspects of the food environment and dietary behavior in studies that do not specifically consider the role of SEP. There is indeed some evidence that the food environment impacts dietary behavior differentially across socioeconomic strata. Three UK studies indicated that having a higher SEP is protective against exposure to unhealthy food environments [25,26,27]. However, evidence for this hypothesis has not been systematically reviewed. A better understanding of how food environments impact high and low SEP groups differentially would contribute to public health strategies targeting dietary inequalities.

Therefore, the aim of this study was to systematically review the evidence for socioeconomic differences in the association between the food environment and dietary behavior. in adolescents and adults. We included studies that stratified their population on the basis of SEP and studied the food environment-diet association in these strata. In addition, we included studies that investigated a single SEP group to assess if associations between the food environment and dietary behavior are generally stronger or more consistent in either high or low SEP populations. 

## 2. Materials and Methods

We conducted a systematic literature review according to the Preferred Reporting Items for Systematic Reviews and Meta-Analyses guidelines [28]. The protocol for this literature search was registered in the Prospero database, registration number CRD42017073587 (can be found via https://www.crd.york.ac.uk/prospero/).

### 2.1. Literature Search

Original, peer-reviewed studies that examined associations between the food environment and dietary behavior in different socioeconomic strata or in a single SEP group were included. Food environments were defined as all objective and perceived aspects of the economic and physical food environment outside the home. Dietary behavior was defined as all measures of dietary behavior of foods and food groups, dietary patterns and food purchasing behavior. Socioeconomic groups were defined as individual, household or area-level measures of education, income, occupation or receiving benefits. Only studies with a study population of adolescents or adults (aged twelve years or over) were considered, as the food choices of children younger than twelve years are less likely to be directly influenced by the food environment (but rather via their parents’ food choices). Furthermore, only studies with an observational study design (including baseline data of experimental studies) were included since we were interested in the differential response to long-term environment rather than the differential response to a (short term) change in the food environment as is the case in experiments. A detailed overview of the inclusion criteria is available in Table 1. A systematic literature search was performed in 4 electronic databases (Medline, Embase, Psycinfo and Web of Science) for studies published up to June 2018 in the English or Dutch language. The search strings can be found in Supplementary File S1. An additional manual search was performed to identify relevant articles based on the reference list of included studies. 

Papers identified by the search strategy were uploaded in Rayyan for screening. Rayyan is a free web and mobile app that facilitates multi-author screening of abstracts and titles [29]. To refine the in- and exclusion criteria, the first 100 retrieved articles were screened on the basis of title and abstract. Inclusion rates were compared and if necessary, adjustments were made to the criteria. Thereafter, titles and abstracts were equally divided among five of the authors for screening of relevance according to the review inclusion criteria. 

Full text versions of all records deemed eligible on the basis of title and abstract were searched through the four electronic data bases or alternatively searched via Google Scholar or requested by e-mail from the corresponding authors. The retrieved full texts were reviewed for inclusion. 

### 2.2. Data Extraction

The following information was extracted from the included studies:

- Study characteristics (author, year of publication, sample size, response rate, country, study design, objective);
- Population characteristics (e.g., age group);
- Type of dietary behavior (e.g., healthy eating index, adherence to dietary guidelines, fruit and vegetable (F & V) intake);
- Aspect of food environment studied (e.g., distance to nearest supermarket);
- Indicator of SEP (e.g., education, income, social class);
- Results and conclusion.

Extracted data was summarized in tables based on type of food environment measure (e.g., perceived food environment, school food environment).

### 2.3. Assessment of Methodological Quality

All included studies were independently assessed for methodological quality using the 14-item NIH quality assessment tool for observational cohort and cross-sectional studies [30]. With regard to the item ‘accuracy, objectivity, validity and reliability of the outcome measures’, studies were rated positively when using a dietary assessment tool that was validated in the population under study or when using objective information on dietary purchases. Studies were rated neutrally when using a previously validated dietary assessment tool or using a combination of self-reported and objective dietary purchase outcomes. Studies were rated negatively when using a non-validated dietary assessment tool, or when a previously validated tool was adapted without further validation. Generally, articles were rated ‘Good’ when they had ≥6 times ‘Yes’, ‘Fair’ when they had 3–5 times ‘Yes’, and ‘Poor’ when they had 0–2 times ‘Yes’, but per instruction of the quality assessment tool, an assessment of the overall quality of the article was also included in the rating.

## 3. Results

**Figure 1:**
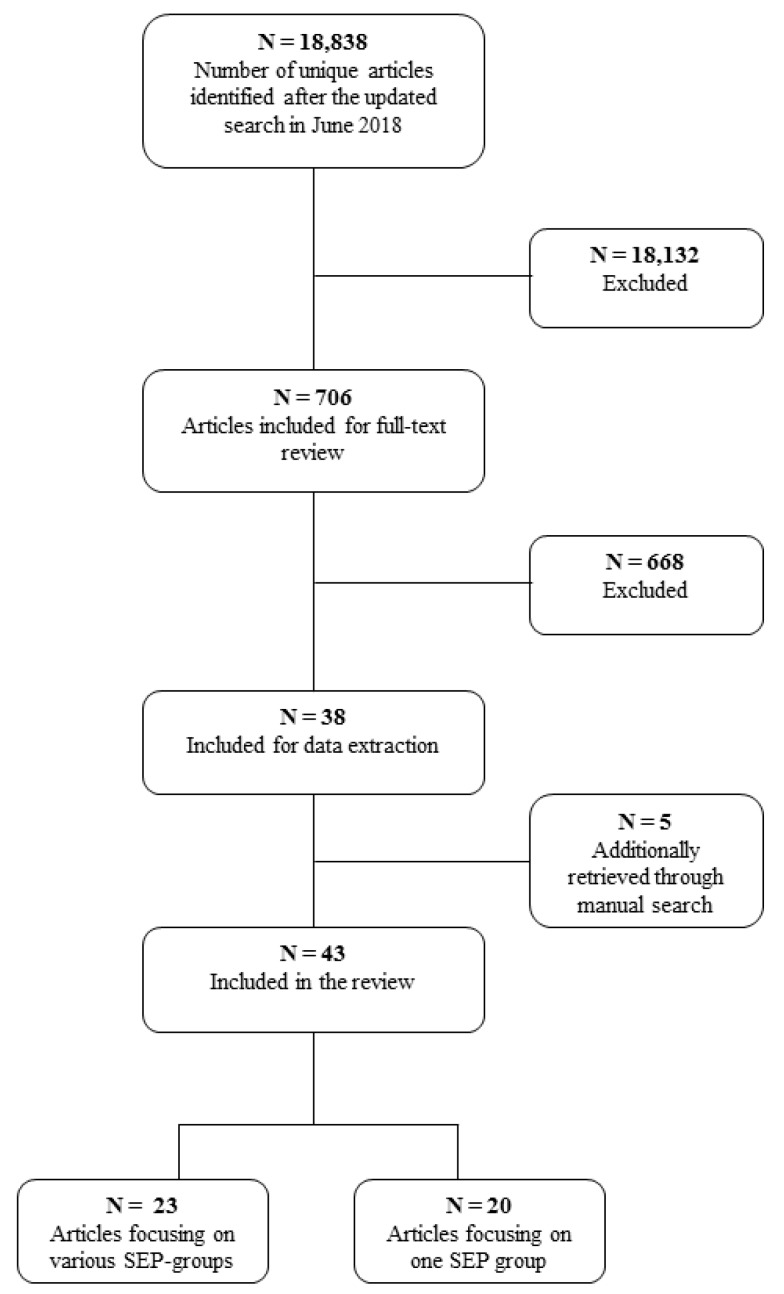
Study selection flow chart.

After removal of duplicates, 18,838 articles were screened on the basis of title and abstract. A total of 18,132 records were excluded after reading title and/or abstract, leaving 706 articles for full-text screening. A further 668 articles were excluded on the basis of inclusion/exclusion criteria. Based on the reference lists of the thirty-eight studies included for data extraction, five additional papers were identified. Most exclusions were done because authors did not present food environment-diet associations by SEP, but only associations between the food environment and dietary behavior adjusted for SEP as covariate. A total of forty-three papers were included in the review, of which twenty-three studied the association between aspects of the food environment and dietary behavior across different SEP strata (Table 2) and twenty studied this association in a single SEP group (Table 3). The study selection flowchart is presented in Figure 1. 

### 3.1. General Study Characteristics

General study characteristics are presented in Table 2 and Table 3. Briefly, the majority of studies were conducted in the USA (n = 27), followed by the UK (n = 5), Brazil (n = 3), Australia (n = 2) and Mexico, New Zealand, Finland, Canada, Hong Kong and France (n = 1 each). Thirty-nine out of forty-three studies had a cross-sectional design. Exceptions were two repeated cross-sectional studies by Colchero et al. [31] and Jilcott Pitts et al. [32] and two longitudinal studies by Meyer et al. [33] and Rummo et al. [34]. Thirty studies were conducted in an adult population [25,32,33,34,35,36,37,38,39,40,41,42,43,44,45,46,47,48,49,50,51,52,53,54,55,56,57,58,59], seven were focused on adolescents [60,61,62,63,64,65,66] and six were conducted in a mixed age population [31,67,68,69,70,71]. Consumption or purchase of F & V were used as measure of dietary behavior in twenty-eight studies [32,34,35,36,37,38,39,40,42,43,44,46,48,49,52,53,55,56,58,60,61,63,64,66,67,68,70,71], seventeen used indicators of unhealthy dietary behavior such as intake or purchase of sugary sweetened beverages, snacks or fast food (FF) [26,31,32,33,38,40,42,48,51,53,58,62,64,65,66,69,70], and eleven used a composite index overall quality or healthfulness of the diet [25,34,38,41,44,45,47,50,54,57,59]. 

### 3.2. Associations of the Food Environment and Dietary Behaviours across Different SEP Strata

Of the twenty-three studies that considered the association between the food environment and dietary behavior interacting with or stratified according to SEP (Table 2), six papers focused on *economic aspects of the food environment*, namely objective measures of food prices [31,33,44,45,46,61]; twelve papers considered objectively measured aspects of the food environment such as *access, proximity and quality of the food environment* [25,26,34,47,48,49,50,51,52,69,70,71]; four studies focused specifically on the *school food environment* [62,63,64,65]; and one study studied the *perceived availability* of foods [66]. Half of the studies were conducted in the USA. SEP indicators used to investigate moderating effects were (household) income [31,34,44,48,63], (parental) education [25,26,51,65], household poverty/deprivation [45,64,66], employment status [70], public versus private schools [62], area level deprivation [49], receiving benefits [69] and a combination of multiple indicators [33,46,47,50,52,61,71]. 

Overall, studies that considered the association between *economic aspects of the food environment* and dietary behavior found differential associations on the basis of SEP. In five studies, objectively measured higher food prices of unhealthy foods were associated with either lower consumption of unhealthy foods or higher consumption of healthier foods, and higher prices of F & V were associated with lower consumption of F & V [31,33,44,46,61]. Four of these studies found that low SEP groups were more responsive to food prices [30,32,45,60]. One study did not find differential effects by SEP when linking fast food prices to fast food consumption but did observe that higher F & V prices were only associated with higher F & V consumption in a low SEP group [44]. The authors speculated that other, unmeasured, competing factors may have led to this unexpected finding [44]. Finally, one study found that a higher SEP group was more responsive to price promotions, most notably price promotions on healthier foods [45].

Studies that examined *objectively measured access, proximity and quality of the food environment* often did not find significant associations with dietary behavior, nor interactions by SEP [47,49,70,71]. Most studies focused on access and proximity of food retailers in the neighborhood. Three studies found associations between these aspects of the food environment and dietary behavior, but without any indication of moderation by SEP [50,52,71]. Four studies reported that associations between access, proximity and quality of the food environment were more strongly associated with dietary behavior in the socioeconomically disadvantaged subgroup compared to the higher socioeconomic groups [25,34,51,69], of which three studies focused on the neighborhood food environment and one on the in-store food environment. That is, these studies showed that less healthful in-store supermarket environments, poorer food environments, a higher proportion of convenience stores and shopping at supercenters or convenience stores were associated with a lower diet quality or unhealthier dietary behavior among those with low SEP, but this association was weaker, non-significant or in the opposite direction among those with high SEP. The authors suggested that fewer individual or neighborhood-level material and psychosocial resources make individuals with low SEP more vulnerable to availability and marketing of unhealthier foods [25,34,51,69]. Finally, one study observed that dietary inequalities between low and high income individuals were only present in neighborhoods with a low density of supermarkets and fresh produce markets [48] and one study reported that educational inequalities in fast food consumption were stronger in areas with higher fast food outlet exposure than in areas with lower fast food exposure [26]. 

All four studies examining socioeconomic differences in the association between the *school food environment* and dietary behavior showed interaction by SEP, although not all in the same direction. Two studies showed that low SEP adolescents benefitted more from healthy options in the school food environment than high SEP adolescents [63,64], one study showed that high SEP adolescents benefitted more from healthy options in the school food environment than low SEP adolescents [62] and one study showed that a fast food outlet or grocery store close to school was associated with irregular eating habits (described as an undesirable behavior) in low SEP adolescents only [65]. 

Finally, one study considered the *perceived food environment* and found that perceived availability of FF outlets, restaurants and convenience stores close to home was associated with unhealthy intakes, with larger effect sizes in adolescents from less affluent families than in adolescents from more affluent families [66]. 

### 3.3. Associations of the Food Environment and Dietary Behaviours in a Single SEP Group

All but one of the twenty studies that reported on the association between the food environment and dietary behavior in a single SEP group (Table 3) focused on a socioeconomically disadvantaged group in terms of receiving benefits, living in a deprived area, having low income, being low educated or having food insecurity status. The exception was the study by Leischner et al. which focused on university college students, thereby focusing on higher educated young adults [41]. Sixteen out of the twenty studies were conducted in the USA. Most of these twenty studies considered more than one aspect of the food environment: fourteen papers considered *availability and quality of stores* in the neighborhood [32,35,38,39,40,41,42,43,53,56,57,59,67,68]; ten papers studied *access, distance or time taken to travel to stores* [32,36,37,40,43,57,58,59,60,67,68]; and seven papers studied *economic aspects of the food environment* such as objective food cost and/or perceived affordability [32,43,53,54,55,56,67]. 

In the studies conducted among a socioeconomically disadvantaged group that considered *availability and quality of stores* in the neighborhood [32,35,38,39,40,41,42,43,53,56,57,59,67,68], five studies observed that perceived [39,40,56] and objective [36,41,42,57] availability of stores selling healthier products was associated with healthier dietary behavior and two studies observed that availability or use of stores selling unhealthier products was associated with unhealthier dietary behavior [42,60]. Six studies found no association between availability in food stores and dietary behavior [32,35,38,43,59,60]. One study showed that perceived food store access was not associated with F & V intake, while having both a supercenter and convenience store nearby was [35]. Another study showed that F & V and SSB consumption was higher in specific food shopping locations [53] but provided no explanation for this finding. 

Of the ten papers that studied *access, distance or time taken to travel to stores* [32,36,37,40,43,57,58,59,60,67,68], six found non-significant associations [32,37,43,58,59,67,68] and seven observed positive significant associations [32,36,37,43,57,59,68]. For example, Rose et al. found that having ‘easy access’ to a supermarket was associated with higher daily fruit use, while perceived travel time was not. No studies reported unexpected associations. 

Of the six papers that studied the role of *economic aspects of the food environment* for dietary behavior [32,43,53,54,55,56], two found no significant associations with objective food prices or perceived costs [32,56], and three found a negative association, such that higher objective food prices, higher perceived food costs and lower self-reported affordability were associated with lower diet quality or lower intake of healthy foods [43,54,55]. One study did not find an association between objective prices of F & V and F & V consumption but did find that higher SSB prices were associated with higher consumption of SSBs [53], which is an unexpected direction of the association. The authors suggested that this finding may be due to insufficient variation in SSB prices or misreporting of SSB consumption [53].

### 3.4. Quality Assessment

Of the forty-three included studies, twenty-six received a ‘good’ rating, fifteen received a ‘fair’ rating and two received a ‘poor’ rating (Table 4). Most studies scored poorly on the sample size justification and most studies did not use a validated tool to measure dietary behavior or used a previously validated tool but did not validate it in their study population. The two studies that received a ‘poor’ rating additionally did not describe their population clearly. 

### 3.5. Results by Study Characteristics

Finally, we assessed whether we found evidence for socioeconomic differences in the association between aspects of the food environment and dietary behavior in subsamples of the included studies. Taking into account study characteristics, different associations between the food environment and dietary behavior across SEP groups were observed in: Seven out of seven studies conducted among adolescents only [60,61,62,63,64,65,66], ten out of fifteen studies conducted outside the USA [25,31,36,45,51,55,60,62,65,66], three out of four non-cross-sectional studies [31,33,34], and fourteen out of twenty-six studies rated as having ‘good’ quality [25,31,33,34,36,40,42,44,46,54,55,57,63,65,69].

## 4. Discussion

The aim of this study was to systematically review the literature on socioeconomic differences in the association between the food environment and dietary behavior of adolescents and adults. We included studies that stratified their population on the basis of SEP as well as studies that considered the association between the food environment and dietary behavior in a single SEP group (e.g., only low-income groups). The included studies were diverse in their measures of the food environment and dietary behavior, indicators of SEP, and their findings. 

We hypothesized that the food environment would have a stronger effect on dietary behavior in those with lower SEP, and that associations between the food environment and diet would be more consistent if only one socioeconomic group was considered. We found some evidence to support the first hypothesis: In the studies that focused on *economic* (n = 6) and *school food* (n = 4) environments, associations with dietary behavior tended to be stronger in the socioeconomically disadvantaged subgroups. However, this was not the case for studies focusing on *objectively measured access, proximity and quality* of the food environment (n = 12). Only one study focused on *perceived food environments*, therefore little can be concluded about the strength of evidence for socioeconomic differences in these types of studies. We did not find strong evidence for the second hypothesis since associations in specific socioeconomic groups (mostly in low SEP groups) were inconsistent, with about half of the studies finding non-significant associations. Studies among adolescents (n = 7) and non-cross-sectional studies (n = 4) generated most consistent results. 

The more consistent evidence for the interaction by SEP for economic and school food environments may be due to the fact that these aspects of the food environment are more delimited and that ‘exposure’ to these aspects of the food environment is easier to define compared to aspects of availability and accessibility in the overall food environment. The significant amount of time (‘exposure’) adolescents spend at school may explain why this type of environment has a relatively consistent influence on dietary behavior. It may be speculated that adolescents with a high SEP have a healthier home food environment, while low SEP with unhealthier home food environments may therefore benefit more from a healthy school food environment [72]. The results for economic aspects of the food environment echo the findings from studies demonstrating a stronger response to tax and subsidy policies from those with lower SEP [73,74]. Future studies could examine the pathways through which these socioeconomic differences arise; we speculated that both material and psychosocial resources may play a role, but literature on these pathways is scarce [75].

In the studies considering a single SEP group, predominantly focused on socioeconomically disadvantaged populations, evidence for an association of the *availability and quality of stores, access, distance or time taken to travel to stores*, and *(perceived) food costs* with dietary behavior was inconsistent. About half of the studies found significant associations in the expected direction, a few found significant associations in an unexpected direction, and the remainder found no significant associations. This is comparable to the findings of systematic literature reviews on the association between the food environment and dietary behavior across socioeconomically diverse populations [14,15,16,17,18,19,20,21,22,23,24], providing little evidence that associations are more consistent when a more socioeconomically homogeneous population is considered. Many of the studies that focused on socioeconomically disadvantaged populations defined their population on the basis of community-level deprivation or income. This may leave room for socioeconomic variability within these communities, particularly if those with higher SEP were more likely to participate in the study. As such, the studies focusing on one specific SEP group may not truly have resulted in studies conducted in a socioeconomically homogeneous group. Additionally, on the basis of this literature review, little can be concluded about the role of the food environment for dietary behavior in a high SEP population, as we only identified one study that focused on such a population. 

On the basis of previous literature reviews [14,15,16,17,18,19,20,21,22,23,24] we speculated that observed null associations in a socioeconomically diverse sample may be due to opposing associations in higher and lower SEP groups, but many studies did not find significant differences between SEP groups. It is likely that the inconsistencies observed in this literature review have similar causes as the inconsistencies observed in general literature reviews on associations between the availability and accessibility of the food environment and diet. Namely: That similar measures of the food environment are difficult to compare between different contexts; that food environments are often simplified to metrics of single types of food retailers (i.e., proximity to supermarkets, or availability of F & V in convenience stores), while the food environment encompasses a broad range of interacting factors (e.g., an interplay of proximity, availability, marketing, labelling, etc.); and that researchers make many assumptions about the places and ways in which food environments influence dietary behavior [20,22]. This may be reflected in our finding that SEP differences were most consistent for studies focusing on economic and school food environments, which represent much more narrow aspects of the food environment than access, availability and quality of food retailers. In general, adherence to reporting guidelines on food environment studies such as the Geo-FERN reporting checklist [76] would facilitate the comparison of such studies in systematic reviews.

### Strengths and Limitations

This is the first systematic literature review that examined socioeconomic differences in the association between the food environment and dietary behavior. Strengths of this study were the broad definition of food environment variables in order to capture all relevant literature; the use of four search engines; the performance of a rigorous quality assessment of the included studies; and the fact that screening, data extraction and quality assessment was performed by at least two researchers each. However, although systematic literature reviews occupy a top position in the hierarchy of evidence, they, including this one, suffer from a number of limitations. Although we piloted the screening process, the involvement of multiple authors in the screening process and the high number of potentially relevant articles in general may have led to the erroneous exclusion of relevant articles. Furthermore, the heterogeneous nature of the included studies prevented us from performing a meta-analysis of the findings, and this hampers the assessment of publication bias: Authors may not have reported non-significant interaction terms with SEP, which may have led to an overestimation of the SEP-differences in this review. The classification of studies into categories of food environment measures may also be noted as a limitation: As studies in single SEP groups examine different aspects of the food environment than studies stratified by SEP we were unable to use the same classification for both types of studies. Finally, whilst there was no limitation for language during the search strategy, our review consists entirely of articles published in English. This could be due to the fact that other relevant articles may not have been indexed in the electronic databases used for this review. 

## 5. Conclusions

Evidence for socioeconomic differences in association between the food environment and dietary behavior was inconsistent, although a limited amount of studies focusing on economic and school food environments generally observed stronger associations in low SEP populations than in high SEP populations. Studies on the association between food environment and dietary behavior in a single SEP group were no more consistent than studies in a mixed population observed in previous literature reviews. As such, it is unlikely that the inconsistencies in the association between the food environment and diet that have been observed thus far are attributable to a differential response to food environments from high and low SEP groups. 

## Figures and Tables

**Table 1:** Inclusion criteria.

Determinant	Criteria
Population	Healthy, non-institutionalized persons of age 12 years and older
Food environment	Objective measures (e.g., geographic information systems) and subjective measures (e.g., perceived food environment) of the food environment outside the home, including but not limited to physical availability or accessibility of food retailers, availability or prices of foods in work, school or shopping environments, quality of stores and food products
Socioeconomic measures	Individual, family or area-level indicators of educational attainment, income, occupational status, or other indicators of socioeconomic position (e.g., food insecurity)
Dietary behavior	Intake of specific foods or food groups, dietary patterns, meeting dietary recommendations, indicators of dietary quality, food choices, or food purchasing behavior
Study design	Observational studies, baseline data of experimental studies

**Table 2:** Overview of included studies reporting on associations between aspects of the food environment and diet across different socioeconomic groups—by type of food environmental factor studied.

Measures								
Author (Year) Country	N	Age Group	Study Design	Study Focus	Food Environment	Dietary Outcome	Indicator of SEP	Summary of Findings
**Economic aspects of the food environment**								
Beydoun et al. (2008) USA [44]	7331	Adults aged 20–65 yrs	CS	Association: Food prices and dietary intake	Price of FF and of F & V based on geocode data of commonly consumed foods	aMED score, HEI score, F & V intake, FF consumption based on 24 h recalls	Poverty-Income-Ratio	Higher FF prices were associated with higher diet quality in all groups. Higher F & V prices were also associated with higher diet quality, and this associated was mostly present among the poor subgroup.
Colchero et al. (2015) Mexico [31]	2006: 19,512; 2008: 27,994; 2010: 25,805 households	Households	Repeated CS	Own and cross price elasticities for soft drinks and SSBs	Price elasticity of SSBs (prices derived from household expenditures using methodology to produce national consumer price index)	Proportion of household expenditures on SSB with respect to total expenditures on food and drinks	Income	SSB and soft drink demand in low income groups was reduced more by higher prices than in high income groups.
Meyer et al. (2014) USA [33]	5115	Adults aged 18–30 yrs	LG: Prospective follow-up 5x over 20 yrs	Association: Price changes and FF consumption	Food prices based on Consumer price data from the Council for Community and Economic Research	FF consumption based on a limited number of questions	Education and income	Larger decrease in FF consumption per unit change in price for those with relatively less education or with lower income.
Nakamura et al. (2015) UK [45]	26,986 households	Adults (48.60 ± 15.84 yrs)	CS	Association: price promotions and food purchases	Promotional pricing of food categories (healthy and less-healthy) from 11 supermarket chains	Sales of healthier and less-healthy versions of foods based on transaction records from a household panel	Household SEP	Higher SEP groups were more responsive than lower SEP groups to promotions for foods, most notably for promotions on healthier foods.
Powell et al. (2009) USA [46]	3739	Adults aged 18–23 yrs	CS	Association: Food prices and F & V intake	Local area food price data. Price index for F & V; meat, dairy & bread; food at home (F & V, meat, dairy, bread); FF Based on American Chamber of commerce price data	2 questions on F & V intake	Educational level, parental education, income	Lower income, lower educated young adults and those with lower educated mothers and lower income parents were more likely to eat fewer F & V when food prices were higher.
Powell et al. (2011) USA [61]	1134	Adolescents aged 12–18 yrs	CS	Association: Food prices and availability of food stores and food consumption patterns	Two food-related price indices: a food at home grocery index and away-from-home FF index. Availability food stores and restaurants. Based on American Chamber of commerce price data	Number of days in the last week that F & V, fruit juice, meat, nonmeat protein, dairy, grains, and sweets were consumed, based on an audio computer-assisted self-interview	Maternal education, working status, family income	Among low income adolescents: higher FF prices were associated with a higher number of days nonmeat protein consumption. Increased supermarket availability was associated with higher frequency vegetable intake. FF restaurant availability was not significantly associated with any of the food consumption patterns.
**Objectively measured access, proximity, quality of food environment**								
Burgoine et al. (2016) UK [26]	5958	Adults aged 29–62 yrs	CS	Association: Fast food outlet exposure and fast food consumption: moderation by educational attainment	Counts of fast food outlets within 1-mile Euclidean buffers around the home and work location	Consumption of energy-dense foods typically obtained from fast food outlets (e.g., pizza, burgers, chips, fried fish, fried chicken) based on a semi-quantitative food frequency questionnaire	Educational attainment	Greater fast food outlet exposure was associated with greater fast food consumption. The difference in fast food consumption between those with lowest and highest education level was strongest in those most exposed to fast food outlets.
Chrisinger et al. (2018) USA [47]	4962	Adults aged 18+ yrs	CS	Association: Trip, store and shopper characteristics with trip HEI scores.	Types of food shops used (conventional supermarket, discount/limited assortment store, natural/gourmet store), distance from shopper’s home to full-service supermarket	HEI-2010 score and consumption of multiple food groups based on reported food purchases at household level	Income/SNAP eligibility, educational level	Shopping in conventional supermarket or natural/gourmet store was associated with higher HEI scores. Spending less money was associated with lower HEI scores. Distance travelled from home was not associated with HEI. Non-SNAP eligible households had higher HEI scores when shopping at convenient supermarkets and discount/limited assortment stores than SNAP households and households that were SNAP eligible but not receiving.
Duran et al. (2014) Brazil [48]	1842	Adults (36.5 ± 11 yrs)	CS	Association: Local retail food environment and consumption of F & V and SSB	Proximity and density of supermarkets and fresh produce markets within 1.6 km buffer from participants’ homes	Consumption of F & V and SSB based on a short number of questions	Income	In neighborhoods with a low density of supermarkets and fresh produce markets, low income individuals had a significantly lower F & V intake than high income individuals. This association disappeared in neighborhoods with a larger number of supermarkets and fresh produce.
Gustafson et al. (2017) USA [69]	2936 households (primary food shoppers)	Households	CS	Association: Neighborhood food store availability and primary food store choice; and primary food store choice and types of food purchases	Availability of food venues within 1 mile of the home: (1) Supermarkets (sells primarily foods); (2) supercenters (food + significant amount other items); (3) convenience stores; (4) combination grocery stores (food + prepared food items + other); (5) medium and large grocery stores	Food purchases of (1) SSB and (2) low-calorie beverages and water based on scanned barcodes on food products; saved store receipts; and information written in a food book	SNAP households or SNAP-eligible households (185% of poverty threshold)	Having supermarkets and supercenters nearby was associated with shopping in supermarkets and supercenters, respectively, but only in SNAP households. Only in non-SNAP households, having grocery stores nearby was associated with shopping there. Shopping at supercenters or convenience stores was associated with higher odds of purchasing SSB. Shopping at supercenters was associated with higher odds of purchasing water/low calorie beverages in both SNAP and non-SNAP households. Shopping at grocery stores was only associated with higher odds of purchasing SSB in SNAP households. Shopping at supermarkets was only associated with higher odds of purchasing water/low calorie beverages in SNAP households.
Jack et al. (2013) USA [49]	15,634	Adults aged 18+ yrs	CS	Association: Density of food outlets and F & V consumption	Density of healthy food outlets and access to healthy food outlets based on zip codes	F & V intake based on short number of questions	Low and high poverty zip-codes	The density of healthy food outlets did not predict consumption of fruits or vegetables in the total sample, the low poverty sample and the high poverty sample.
Macdonald et al. (2011) Scotland [70]	1149	Adolescents and adults aged 16+ yrs	CS	Association: Proximity to food retail stores and dietary patterns	Proximity to general stores, F & V stores and supermarkets using GIS data in 500 m and 1 km buffers	Intake of F & V and high fat snacks based on limited number of items in survey	Car ownership and employment	Few significant associations between proximity to food outlets and F & V intake or high fat snacks intake were observed. The borderline significant association between living near a supermarket and not eating F & V regularly was not different between employed and unemployed adults, but did differ between those with and without a car. That is, those with a car had borderline significant higher odds of consuming F & V regularly when a supermarket was present within 1 km.
McInerney et al. (2016) Canada [50]	446	Adults aged 21+ yrs	CS	Association: Neighborhood food environment and diet quality	Objective measures of food destination presence, density and diversity within walkshed of 400 m from participants’ homes	Canadian adapted Healthy Eating Index (C-HEI) based on FFQ data	Education and income	A higher the number of food destinations within 400 m of home, regardless of type, was associated with higher C-HEI scores. No statistically significant interactions between walkshed food environment variables and socioeconomic status in relation to the C-HEI.
Pearce et al. (2008) New Zealand [71]	12,529	Adolescents and adults aged 15+ yrs	CS	Association: Neighborhood accessibility to supermarkets and convenience stores and F & V consumption	Access to supermarkets and convenience stores based on travel time along the road network using GIS	Eating recommended F & V levels based on limited number of items in survey	Education, social class, employment and income	No association was observed between neighborhood access to supermarkets or convenience stores and the consumption of F & V. Better access to convenience stores was associated with lower vegetable consumption. None of the interaction effects between access to convenience stores and any of the socioeconomic variables were significant.
Rummo et al. (2015) USA [34]	3299	Adults (25.0 ± 3.6 yrs)	LG	Association: Neighborhood convenience stores and diet quality	Convenience store relative to total food outlets based on a 3 km buffer around participants’ homes	A priori diet quality score; beneficial foods (whole grains, F & V); adverse foods (SSB, ASB, salty snacks, processed meats, desserts) based on an FFQ	Individual-level income	A higher proportion of convenience stores relative to total food stores/restaurants was associated with lower diet quality scores and this association was stronger among low income participants. For specific food groups; only whole grain consumption was negatively associated with the % neighborhood convenience stores relative to total food stores/restaurants, and this association was also stronger among low income participants.
Vogel et al. (2016) UK [25]	829	Adults (31.78 ± 6 yrs)	CS	Association: In-store supermarket environment and maternal dietary quality	Composite score representing the healthfulness of the in-store supermarket environment	Prudent dietary pattern score based on a 20-item FFQ	Educational attainment	A strong positive relationship between dietary quality and store healthfulness was observed among low educated mothers, but no significant association among mid educated mothers; and poorer store healthfulness was associated with better dietary quality among high educated mothers.
Vogel et al. (2017) UK [51]	838	Adults (31.78 ± 6 yrs)	CS	Association: Overall food environment and maternal dietary quality	The balance between healthy and unhealthy food stores in 1000 m buffers using GIS data	Prudent dietary pattern score based on a 20-item FFQ	Educational attainment	Poorer food environments were associated with higher diet quality scores among high educated mothers and (non-significant) lower diet quality scores among low educated mothers.
Zenk et al. (2009) USA [52]	919	Adults (46.28 ± 0.84 yrs)	CS	Association: Residential neighborhood retail food environment and F & V intake	Observed F & V availability, variety, quality, affordability within ½ mile Euclidean distance from the center of a residential block	F & V intake using an FFQ	Education, income, employment	There was no evidence that individual sociodemographic characteristics moderated the relationship between the neighborhood food environment and F & V intake.
**School food environment**								
Azeredo et al. (2016) Brazil [62]	109,104	Adolescents aged 11+ yrs	CS	Association: Food environment in public and private schools and in the immediate surroundings and the consumption of unhealthy food	Availability of healthy/unhealthy foods in school cafeteria or nearby school, reported by school principal. Provision of Brazilian school food program in public schools	Consumption of soft drinks, deep fried salty snacks, bagged salty snacks and sweets based on a validated questionnaire	Public vs. private schools	The presence of cafeteria selling fruit was negatively associated with the consumption of salty snacks in private schools only. Other differences were not statistically significant. Eating foods from the school food programme was associated with lower purchasing of unhealthy foods, but only in public schools.
Longacre et al. (2014) USA [63]	1542	Adolescents (14.4 ± 1.04 yrs)	CS	Association: F & V intake while school was in session (exposed to school food) and when school was not in session (not exposed to school food)	Exposure to school food based on timing of survey (summer months vs. school year)	F & V intake based on a 2-item measure from the Youth Risk Behavior Surveillance System	Household income	Among adolescents unexposed to school food, household income and F & V intake was positively associated. Among adolescents exposed to school food, F & V intake was similar across income categories. Interaction analysis indicated that adolescents in the lowest income category had higher F & V intake if they obtained school food, and adolescents in the higher income category had lower F & V intake if they obtained school food. The results indicate that exposure to school food mitigates income-related disparities in adolescent F & V intake, and that this mitigation is beneficial for low-income students.
Vericker et al. (2013) USA [64]	5530	Adolescents	CS	Association: Competitive food and beverage availability in school and F & V and SSB intake	Foods and beverages offered at school that compete with the National School Lunch Program	F & V and SSB intake based on food frequency questionnaires	Family poverty status	Competitive food access was not associated with F & V intake and SSB intake. Only adolescents from families with incomes below the poverty line had lower F & V consumption if they lost access to competitive foods.
Virtanen et al. (2015) Finland [65]	23,182	Adolescents (15.4 ± 0.63 yrs)	CS	Association: Proximity to FF outlets and grocery stores to school and eating habits	Distance to a food outlet 100, 100–500 and >500 m from school entrance	Skipping free school lunch, obtaining snacks outside of school based on an unknown number of survey items	Parental education	A FF outlet or grocery store close to school was associated with irregular eating habits, but with an accumulation of irregular eating behavior in low-SEP adolescents only. Proximity to a food outlet was associated with higher odds of skipping school lunch in high SEP adolescents.
**Perceived food environment**								
Ho et al. (2009) Hong Kong [66]	34,369	Adolescents (14.5 ± 0.11 yrs)	CS	Association: Perceived availability of food stores and intake of F & V, SSB and junk foods	Perceived availability of FF shops, restaurants and convenience stores within 5 min walking distance from home	Intake of F & V, high fat foods and junk food/SSB based on four questions on frequency of consumption	Perceived family affluence	Perceived availability of FF shops, restaurants, and convenience stores were associated with unhealthy dietary intakes. This was stronger in boys from less affluent families.

CS = cross-sectional. FF = fast food. FFQ = food frequency questionnaire. F & V = fruit and vegetables. Hr = hour. Km = kilometer. LG = longitudinal. SSB = sugar sweetened beverage. Yrs = years.

**Table 3:** Overview of included studies reporting on associations between aspects of the food environment and diet in a single socioeconomic group – in alphabetical order.

Measures								
Author (Year) Country [Ref]	N	Age Group	Study Design	Study Focus	Food Environment	Dietary Outcome	Indicator of SEP	Summary of Findings
Basu et al. (2016) USA [54]	14313	Adults	CS	Association: County-level cost of food and dietary quality	Regional price parity relative to national average: food costs, area cost of living and cost of rent	HEI-2010 score and acquisition of specific food groups based on national household food acquisition data	SNAP participation, educational level, employment status, household income, rent/ mortgage	Higher food cost was associated with lower volume of acquired F & V and whole grains; with significantly greater acquisitions of refined grains, dairy products, protein, fats and oils, and added sugars; and with lower overall HEI scores.
Bihan et al. (2010) *France* [55]	295	Adults (44.8 ± 8.2 yrs)	Baseline data of intervention study	Association: Affordability of F & V and F & V intake	Self-reported affordability of F & V in the local area/where people shop	Frequency of F & V intake based on a 16-item questionnaire	Individual deprivation level (composite score)	Participants who reported not being able to afford F & V had lower F & V intake frequency.
Blitstein et al. (2012) *USA* [56]	526	Adults aged 18–75 yrs	CS	Association: Shopping at supermarkets, farmer’s markets and coops, perceived costs and F & V intake	Self-reported F & V shopping environment (supermarket vs. farmer’s market or coop). Perception of cost F & V	F & V intake based on a 4-item questionnaire	Participation in assistance programmes (including SNAP)	Participants shopping at coop/farmer’s market were more likely to eat ≥3 servings F & V. No association between perceived cost and F & V intake was observed.
Camacho-Rivera et al. (2016) *USA* [38]	362	Adults	CS	Association: Perceptions of the neighborhood food environment and presence of foods in the home	Perception of neighborhood food environment (quality and ability to purchase food locally)	Presence of F & V, cheese, dairy, meats, fish, snack foods, cereals, candy, condiments and SSB based on a Home Food Inventory. Weekly frequency of FF intake	Living in public housing or recipient of housing choice voucher program	Residents’ perceptions of the neighborhood food environment were not associated with F & V or SSB presence within the home, or with FF consumption.
Chang et al. (2015) *USA* [67]	237	Households	CS	Association: Travel time to stores selling F & V and F & V intake	Self-reported travel time to purchase F & V. Quality and affordability of F & V	F & V Intake using survey data	Participants of WIC or SNAP, household income	No significant associations between environmental factors and intake of F & V were observed.
D’Angelo et al. (2011) *USA* [59]	175	Adults aged 16–90 yrs Adults (45.7 ± 13.6 yrs)	CS	Association: Access and travel time to food sources and healthy and unhealthy food-getting scores	Self-reported food source—supermarket, corner store, other. Access (walking/car) and travel time to food source	Healthy and unhealthy food-getting scores based on the frequency of obtaining a number of different foods	African American households in 2 low income neighborhoods	Unhealthy food-getting scores were significantly higher for corner store shoppers compared with supermarket shoppers, and for walkers compared with those using all other forms of transportation. Healthy food-getting scores did not differ significantly by main type of food source or transportation
Dubowitz et al. (2015) *USA* [57]	1372	Adults	CS	Association: Food access and purchasing practices	Distance and access (driving) to food shopping outlet based on street network distance. Type of store visited. Audit of in-store marketing and healthy food availability of most commonly used stores	HEI-2005 score based on an automated self-administered 24 h recall	Low income neighborhoods	Distance to the nearest full-service supermarket was not associated with food expenditure. Greater distance to where respondents actually did their major food shopping was associated with lower spending. Distance to the nearest full-service supermarket, distance to major food shopping and driving or getting a ride to food shopping was not associated with HEI scores. Shopping at a specialty store, but not shopping at Superright, Wholesale club, discount grocery stores and meat/seafood markets, was associated with higher HEI scores.
Gase et al. (2014) *USA* [58]	1503	Adults (35.6 ± 12.5 yrs)	CS	Association: Self-reported time and distance to the nearest retail grocery store and healthy and unhealthy food consumption	Self-reported distance to nearest grocery store, time taken to travel to grocery store	Daily intake of F & V (servings) and frequency of SSB intake based on a limited number of questions	Multi-ethnic clients of city health clinics in low income areas; educational level	Neither distance nor time were associated with F & V and SSB intake.
Gase et al. (2016) *USA* [39]	1503	Adults (35.6 ± 12.5 yrs)	CS	Association: Perceived food environment and F & V intake	Perceived availability of fresh F & V in neighborhood	Daily F & V intake based on a limited number of questions	Multi-ethnic clients of city health clinics in low income areas; educational level	The perceived food environment was significantly and positively related to F & V consumption.
Gustafson et al. (2011) *USA* [35]	187	Adult women aged 40–60 (51 ± 7.4) yrs	CS	Association: Perceived and objective measures of the food store environment and F & V consumption	Store level: (i) Objective availability of healthy foods in stores where participants shop; and (ii) perception of availability of healthy foods in stores. Neighborhood-level; (i) measured number & type of food stores within the census tract; (ii) perceived availability of healthy foods	F & V intake based on a validated, rapid food survey	Incomes at or below 250% of the federal poverty level	No association between perceived availability of healthy foods and F & V intake was observed. Residents of neighborhoods with supercenters (healthy food store) had lower consumption of F & V.
Jilcott Pitts et al. (2015) *USA* [40]	205	Adults	CS	Association: Barriers to and facilitators of shopping at farmers’ markets and F & V, SSB and FF consumption	Self-reported farmer’s markets—shopping frequency, shopping at various markets throughout the county, awareness and access to markets; barriers/facilitators of use of farmers’ markets	F & V, SSB and FF consumption based on a validated short FFQ	SNAP recipients	People who ever shopped at farmer’s markets had higher intakes of F & V, and lower intakes of SSB FF.
Jilcott Pitts et al. (2016) *USA* [53]	342	Adults	CS	Association: Primary food store, food prices in those stores and F & V and SSB consumption	Primary food store (out of the 5 stores that were located within 5 miles of a new supermarket), objective food prices of F & V and SSB	F & V consumption based on the validated National Cancer Institute Fruit and Vegetable Screener, SSB consumption was based on an adapted version of the Behavioral Risk Factor Surveillance System	Low income communities	The primary food shopping location was associated with F & V and SSB consumption. Prices of F & V were not associated with F & V consumption. Higher SSB prices were associated with higher SSB consumption.
Jilcott Pitts et al. (2018) *USA* [32]	78–172 depending on location and year	Adults	Repeated CS before and after a new supermarket opening	Association: Distance to primary food store and mean prices of F & V and SSB with consumption of these foods. (Also: Effects of supermarket opening and diet)	Inventory of a representative sample of grocery stores/supermarkets Assessment of F & V and SSB availability and price. Distance from participants’ homes to store location. Perceived access to F & V	F & V consumption based on a F & V screener. Frequency of SSB intake based on questions from the behavioral risk factor surveillance system	Low income communities	Distance and F & V consumption were significantly and inversely associated (even when accounting for prices of F & V and SSB). No other significant associations observed (no changes in diet with the introduction of a new supermarket).
Leischner et al. (2018) *USA* [41]	9790	1st and 2nd year university college students	CS	Association: Availability of more healthful versus less healthful food items in the campus dining hall and food purchases	The availability of entrées in the college campus restaurant, categorized into more healthful and less healthful (list obtained from the campus dining provider)	Purchase of more healthful and less healthful entrée items based on purchases registered through student ID cards	Students in tertiary education	The proportion of more healthful entrée items (15%) corresponded to the purchase of more healthful entrée items (8.0% in fall and 8.9% in spring), and the proportion of less healthful entrée items (85%) corresponded to the purchase of less healthful entrée items (92.0% in fall and 91.1% in spring).
Menezes et al. (2016) *Brazil* [36]	3414	Adults aged 20+ (56.7 ± 8) yrs	CS	Association: Access to healthy food stores and F & V consumption	Within 1600 m buffers around a Health Academy Program (HAP) center. Location, proximity, density and type of commercial food store. Observation tool to derive “healthy food store index”	Frequency and quantity of F & V consumption and preparation methods based on a limited number of questions	Health Academy Program (HAP) users—low educated, low income	A positive relationship between the healthy food store index and F & V intake was observed.
Rose et al. (2004) *USA* [37]	963	Adults	CS	Association: Food store access and F & V consumption	Self-reported distance and access to supermarket (combination score of supermarket shopping, travel time and car ownership variables)	Daily fruit use and household vegetable use based on unknown number of items in a survey database	Food stamp recipients	Living > 5 miles away from principal food store was associated with lower daily fruit use. Having ‘easy access’ to a supermarket was associated with higher daily fruit use. These variables were not associated with daily use of vegetables. Travel time <30 min was not associated with daily use of fruits or vegetables.
Stephens et al. (2011) *Australia* [60]	1014	Adolescents aged 12–15 yrs	CS	Association: Availability of energy-dense foods and F & V intake	Self-reported presence of energy-dense food outlets in neighborhood. Perception of school canteen (incl. quality, price of food)	Frequent intake of F & V (defined as 2x per day vegetables; 1x per day fruit) based on a limited number of questions	Maternal education level	Neighbourhood availability of energy-dense food was associated with lower odds of frequent intake of vegetables (in boys only). No association with perception of school canteen were observed.
Strome et al. (2016) *USA* [68]	1200 households	Households	CS	Association: Access to supermarkets and grocery stores and F & V consumption	Food deserts defined on basis of census tracts including at least 500 individuals, or 1/3 of the census tract’s population residing >1 one mile from a supermarket or grocery store. Self-reported distance from F & V purchase point; mode of transport; expensiveness; availability	Frequency of F & V intake based on a limited number of questions	SNAP and SNAP-eligible households. Educational level. Food security	No association between store proximity and F & V intake was observed. Car ownership was associated with higher vegetable intake in both food insecure and secure participants.
Vaughan et al. (2017) *USA* [42]	1372	Adults	CS	Association: Characteristics and use of food stores and consumption of SSB, added sugars, discretionary fats and F & V	Food desserts – frequency of shopping in different food stores. Audit of food stores	Kcal from SSB, teaspoons of added sugars, grams of discretionary (solid) fats and cups of F & V based on 24 h recalls	Low income neighborhood, household annual income	Shopping more frequently at convenience stores was associated with greater consumption of added sugars; buying food more often at neighborhood stores predicted significantly greater intake of SSBs and discretionary fats (e.g., butter); and buying food more often at supercenters was significantly associated with greater intake of discretionary fats. Conversely, shopping more often at specialty grocery stores and F & V stores was significantly associated with greater F & V consumption.
Williams et al. (2010) *Australia* [43]	335	Adult women aged 18–65 (49.5 ± 10.8) yrs	CS	Association: Perceived availability of foods and F & V consumption	Self-reported access, availability of healthy food and cost of F & V. Objective availability (distance from residence) and accessibility (number within 2 km buffer) of supermarket/F & V shop	Servings of F & V per day (high consumers defined as >2 servings of fruit; >3 servings of vegetables) based on a limited number of questions	Educational level	Perceived cost of F & V was associated with lower odds of high intake. Perceived availability and accessibility was associated with higher odds of high intake. None of the objective measures were associated with F & V intake.

CS = cross-sectional. FF = fast food. FFQ = food frequency questionnaire. F & V = fruit and vegetables. Hr = hour. Km = kilometer. LG = longitudinal. SSB = sugar sweetened beverage. Yrs = years.

**Table 4:** Quality Assessment of included articles.

Author(s)	Year	Objective Clearly Stated	Population Clearly Specified	Participation Rate ≥ 50%	Similar Populations	Sample Size Justification	Exposure Assessed Prior to Outcome Measurement	Sufficient Time Frame	Different Levels of Exposure	Exposure Measures Clearly Defined	Exposure(s) Assessed More Than Once over Time	Outcome Measure(s) Validated and Clearly Defined	Outcome Assessors Blinded	Follow Up Rate	Adjusted for Confounding Variables	Overall Quality
Azeredo et al.	2016	+	+	+	+	-	-	-	NA	-	NA	+/-	NA	NA	+	Fair
Basu et al.	2016	+	+	□	+	-	-	-	+	+	NA	+/-	NA	NA	+	Good
Beydoun et al.	2008	+	+	□	+	-	-	-	-	+	NA	+/-	NA	NA	+	Good
Bihan et al.	2010	+	+	+	+	-	-	-	+	+	NA	-	NA	NA	+	Good
Blitstein et al.	2012	+	-	□	+	-	-	-	-	-	NA	-	NA	NA	+	Poor
Burgoine et al.	2016	+	+	□	+	-	-	-	-	+	NA	-	NA	NA	+	Fair
Camacho-Rivera et al.	2015	+	+	-	+	-	-	-	-	+	NA	+/-	NA	NA	+	Good
Chang et al.	2015	+	-	-	-	-	-	-	-	-	NA	-	NA	NA	-	Poor
Colchero et al.	2015	+	+	+	+	-	-	-	NA	+	NA	+	NA	NA	+	Good
Chrisinger et al.	2018	+	+	□	+	-	-	-	+	+	NA	+/-	NA	NA	+	Good
D’Angelo et al.	2011	+	+	□	+	-	-	-	+	-	NA	-	NA	NA	+	Fair
Dubowitz et al.	2015	+	+	+	+	-	-	-	+	+	NA	+/-	NA	NA	+	Good
Duran et al.	2014	+	+	□	+	-	-	-	+	+	NA	+/-	NA	NA	+	Good
Gase et al.	2014	+	+	+	+	-	-	-	+	-	NA	-	NA	NA	+	Fair
Gase et al.	2016	+	+	+	+	-	-	-	-	-	NA	-	NA	NA	+	Fair
Gustafson et al.	2011	+	+	+	+	-	-	-	+	+	NA	+/-	NA	NA	+	Good
Gustafson et al.	2017	+	-	□	+	-	-	-	+	+	NA	+/-	NA	NA	+	Good
Ho et al.	2009	+	+	+	+	-	-	-	-	-	NA	-	NA	NA	+	Fair
Jack et al.	2013	+	+	+	+	-	-	-	+	+	NA	-	NA	NA	+	Good
Jilcott Pitts et al.	2015	+	+	+	+	-	-	-	-	+	NA	+/-	NA	NA	+	Good
Jilcott Pitts et al.	2016	+	+	□	+	-	-	-	-	+	NA	+/-	NA	NA	+	Good
Jilcott Pitts et al.	2018	+	+	□	+	-	+	+	-	+	+	+/-	NA	-	+	Good
Leischner et al.	2018	+	+	□	+	-	-	-	+	-	NA	+	NA	NA	-	Fair
Longacre et al.	2014	+	+	+	+	-	-	-	+	+	NA	-	NA	NA	+	Good
Macdonald et al.	2011	+	+	+	+	-	-	-	-	+	NA	-	NA	NA	+	Good
McInerney et al.	2016	+	+	-	+	-	-	-	+	+	NA	+/-	NA	NA	+	Good
Menezes et al.	2016	+	+	+	+	-	-	-	+	+	NA	-	NA	NA	+	Good
Meyer et al.	2014	+	+	+	+	-	+	+	+	+	+	-	NA	+	+	Good
Nakamura et al.	2015	+	+	+	+	-	-	-	NA	-	NA	+/ -	NA	NA	+	Fair
Pearce et al.	2008	+	+	-	+	-	-	-	+	+	NA	-	NA	NA	+	Good
Powell et al.	2009	+	+	+	+	-	-	-	+	+	NA	-	NA	NA	+	Good
Powell et al.	2011	+	+	□	+	-	-	-	-	+	NA	-	NA	NA	+	Fair
Rose et al.	2004	+	+	+	+	-	-	-	-	-	NA	-	NA	NA	+	Fair
Rummo et al.	2015	+	+	+	+	-	-	+	+	+	+	+/-	NA	+	+	Good
Stephens et al.	2011	+	+	-	+	-	-	-	+	-	NA	-	NA	NA	+	Fair
Strome et al.	2016	+	+	□	+	-	-	-	-	-	NA	-	NA	NA	+	Fair
Vaughan et al.	2017	+	+	+	+	-	-	-	-	+	NA	+/-	NA	NA	+	Good
Vericker et al.	2013	+	+	□	+	-	+	+	+	-	+	-	NA	-	+	Fair
Virtanen et al.	2015	+	+	+	+	-	-	-	+	+	NA	-	NA	NA	+	Good
Vogel et al.	2016	+	+	□	+	-	-	-	+	+	NA	-	NA	NA	+	Good
Vogel et al.	2017	+	+	-	+	-	-	-	-	+	NA	-	NA	NA	+	Fair
Williams et al.	2010	+	+	-	+	-	-	-	+	+	NA	-	NA	NA	NA	Fair
Zenk et al.	2009	+	+	□	+	-	-	-	-	+	NA	+/-	NA	NA	+	Good

N.B. ‘+’ stands for a positive evaluation; ‘-‘ stands for a negative evaluation; ‘+/-’ stands for a neutral evaluation; ‘□’ means the information was not provided/found in the article; NA = not applicable.

## Supplementary Materials

The following are available online at https://www.mdpi.com/2072-6643/11/9/2215/s1, File S1: Search strings used for the systematic review on ‘A systematic review on socioeconomic differences in the association between the food environment and dietary behaviors’.
