# Supplementary material for: A Systematic Review on Socioeconomic Differences in the Association between the Food Environment and Dietary Behaviors
Source: Nutrients. 2019 Sep 13;11(9):2215. doi: 10.3390/nu11092215 (PMC6769523; doi:10.3390/nu11092215)
Supplement: Supplementary file 1 [file nutrients-11-02215-s001.pdf]

Supplementary Files

Supplementary File S1. Search strings used for the systematic review on 'A systematic review on socioeconomic differences in the association between the food environment and dietary behavior'.

	**Ovid MEDLINE(R) Epub Ahead of Print, In-Process & Other Non-Indexed Citations, Ovid MEDLINE(R) Daily and Ovid MEDLINE(R) <1946 to Present>**<br>**Search date: 4 June 2018**
#	**Searches**
1	exp diet/ or feeding behavior/ or food preferences/ or fast foods/ut
2	(((food? or fruit? or vegetable? or beverage? or softdrink? or soda? or fat or meat or fish or snack? or caloric or nutrition*) adj3 (intake or purchas*)) or ((food? or feeding or eat* or diet* or fruit? or vegetable? or beverage? or softdrink? or soda? or fat or meat or fish or snack? or nutrition*) adj3 (Behavior? or pattern? or habit? or attitude? or preference?))).ab,kf,ti.
3	(((diet* or eating or food or nutrition*) adj5 (prudent or western or mediterranean or DASH or index or quality or pattern or score)) or recommendation compliance index or Energy density score).ab,kf,ti.
4	(health behavior or life style or ((recommendation or adherence) adj3 (food or vegetable? or fruit? or diet*))).ab,kf,ti.
5	or/1-4 [feeding pattern]
6	food supply/ or residence characteristics/ or geographic information systems/
7	(food environment or food destination? or food suppl* or food desert? or food polic* or ((food? or fruit? or vegetable?) adj7 (availab* or access*)) or (food adj3 (secur* or insecur*)) or ((home environment or gis or geographic information systems) and (food or vegetable? or fruit?)) or built environment or neighbo?rhood environment or environmental factor? or catering service?).ab,kf,ti.
8	or/6-7 [food environment]
9	exp socioeconomic factors/
10	(socioeconomic* or socio-economic* or afford* or poverty or social class or social status or social background or high income or low income or (standard adj3 living) or education or educational status or employment or income or occupation* or poverty or economic factor? or ((food or vegetable? or fruit?) adj3 (expens* or price or purchas* or consumption*))).ab,kf,ti.
11	or/9-10 [SES]
12	and/5,8,11

	**Ovid Embase Classic+Embase 1947 to 2018 June 01**<br>**Search date: 4 June 2018**
#	**Searches**
1	feeding behavior/ or food preference/ or diet/
2	(((food? or fastfood or fruit? or vegetable? or beverage? or softdrink? or soda? or fat or meat or fish or snack? or caloric or nutrition*) adj3 (intake or purchas*)) or ((food? or feeding or eat* or diet* or fruit? or vegetable? or beverage? or softdrink? or soda? or fat or meat or fish or snack? or nutrition*) adj3 (Behavior? or pattern? or habit? or attitude? or preference?))).ab,kw,ti.

3	(((diet* or eating or food or nutrition*) adj5 (prudent or western or mediterranean or DASH or index or quality or pattern or score)) or recommendation compliance index or Energy density score).ab,kw,ti.
4	(health behavior or life style or ((recommendation or adherence) adj3 (food or vegetable? or fruit? or diet*))).ab,kw,ti.
5	or/1-4 [feeding pattern]
6	(food/ and desert/) or food availability/ or food security/ or food insecurity/ or catering service/
7	(food environment or food destination? or food suppl* or food desert? or food polic* or ((food? or fruit? or vegetable?) adj7 (availab* or access*)) or (food adj3 (secur* or insecur*)) or ((home environment or gis or geographic information systems) and (food or vegetable? or fruit?)) or built environment or neighbo?rhood environment or environmental factor? or catering service?).ab,kw,ti.
8	((food or fruit) and policy).hw.
9	or/6-8 [food environment]
10	exp socioeconomics/ or income/ or exp social status/ or economic aspect/
11	(socioeconomic* or socio-economic* or afford* or poverty or social class or social status or social background or high income or low income or (standard adj3 living) or education or educational status or employment or income or occupation* or poverty or economic factor? or ((food or vegetable? or fruit?) adj3 (expens* or price or purchas* or consumption*))).ab,kw,ti.
12	or/10-11
13	and/5,9,12

	**Ovid PsycINFO <1806 to May Week 4 2018>**<br>**Search date: 4 June 2018**
#	**Searches**
1	(food/ or fast food/) and (social environments/ or home environment/ or poverty areas/ or rural environments/ or suburban environments/ or towns/ or urban environments/ or neighborhoods/)
2	(food environment or food destination? or food desert? or food polic*).ab,id,ti.
3	((food? or feeding or eat* or diet* or fruit? or vegetable? or beverage? or softdrink? or soda? or fat or meat or fish or snack? or nutrition*) and built environment).ab,id,ti.
4	(((food? or feeding or eat* or diet* or fruit? or vegetable? or beverage? or softdrink? or soda? or fat or meat or fish or snack? or nutrition*) adj15 (area or neighborhood? or environment*)) and (socioeconomic* or socio-economic* or afford* or poverty or social class or social status or social background or high income or low income or (standard adj3 living) or education or educational status or employment or income or occupation* or poverty or economic factor? or ((food or vegetable? or fruit?) adj3 (expens* or price or purchas* or consumption*)))).ab,id,ti.
5	or/1-4 [food environment]

	**Web of Science Core Collection**<br>**Search date: 4 June 2018**

	TS=(((food? or fruit? or vegetable? or beverage? or softdrink? or soda? or fat or meat or fish or snack? or caloric or nutrition*) NEAR/2 (intake or purchas*)) or ((food? or feeding or eat* or diet* or fruit? or vegetable? or beverage? or softdrink? or soda? or fat or meat or fish or snack? or nutrition*) NEAR/2 (Behavior? or pattern? or habit? or attitude? or preference?)))
	TS=(((diet* or eating or food or nutrition*) NEAR/4 (prudent or western or mediterranean or DASH or index or quality or pattern or score)) or recommendation compliance index or Energy density score)
	TS=(health behavior or life style or ((recommendation or adherence) NEAR/2 (food or vegetable? or fruit? or diet*)))
	#1 OR #2 OR #3
	TS=(food environment or food destination? or food suppl* or food desert? or food polic* or ((food? or fruit? or vegetable?) NEAR/6 (availab* or access*)) or (food NEAR/2 (secur* or insecur*)) or ((home environment or gis or geographic information systems) and (food or vegetable? or fruit?)) or built environment or neighbo?rhood environment or environmental factor? or catering service?)
	TS=(socioeconomic* or socio-economic* or afford* or poverty or social class or social status or social background or high income or low income or (standard NEAR/2 living) or education or educational status or employment or income or occupation* or poverty or economic factor? or ((food or vegetable? or fruit?) NEAR/2 (expens* or price or purchas* or consumption*)))
	#4 AND #5 AND #6
